# Molecular epidemiology of peste des petits ruminants virus emergence in critically endangered Mongolian saiga antelope and other wild ungulates

**DOI:** 10.1093/ve/veab062

**Published:** 2021-06-25

**Authors:** Camilla T O Benfield, Sarah Hill, Munkduuren Shatar, Enkhtuvshin Shiilegdamba, Batchuluun Damdinjav, Amanda Fine, Brian Willett, Richard Kock, Arnaud Bataille

**Affiliations:** Department of Pathobiology and Population Sciences, The Royal Veterinary College, Hatfield, AL9 7TA UK; Department of Pathobiology and Population Sciences, The Royal Veterinary College, Hatfield, AL9 7TA UK; Department of Veterinary Services of Dundgobi province, General Authority for Veterinary Services of Mongolia (GAVS), Mandalgobi, Dundgobi Province 4800 Mongolia; Wildlife Conservation Society, Mongolia Program, Post Office 20A, PO Box 21 Ulaanbaatar 14200, Mongolia; State Central Veterinary Laboratory, Ulaanbaatar 17024, Mongolia; Health Program, Wildlife Conservation Society, Bronx, New York 10460, USA; MRC-University of Glasgow Centre for Virus Research, Henry Wellcome Building, Garscube Glasgow, G61 1QH UK; Department of Pathobiology and Population Sciences, The Royal Veterinary College, Hatfield, AL9 7TA UK; CIRAD, UMR ASTRE, F-34398 Montpellier, France; ASTRE, University of Montpellier, CIRAD, INRAE, F-34398 Montpellier, France

**Keywords:** peste des petits ruminants virus, PPR, saiga antelope, PPRV host range, PPRV emergence, PPRV molecular epidemiology, wildlife-livestock interface, PPR Global Eradication Programme, Phylogeography

## Abstract

Peste des petits ruminants virus (PPRV) causes disease in domestic and wild ungulates, is the target of a Global Eradication Programme, and threatens biodiversity. Understanding the epidemiology and evolution of PPRV in wildlife is important but hampered by the paucity of wildlife-origin PPRV genomes. In this study, full PPRV genomes were generated from three Mongolian saiga antelope, one Siberian ibex, and one goitered gazelle from the 2016–2017 PPRV outbreak. Phylogenetic analysis showed that for Mongolian and Chinese PPRV since 2013, the wildlife and livestock-origin genomes were closely related and interspersed. There was strong phylogenetic support for a monophyletic group of PPRV from Mongolian wildlife and livestock, belonging to a clade of lineage IV PPRV from livestock and wildlife from China since 2013. Discrete diffusion analysis found strong support for PPRV spread into Mongolia from China, and phylogeographic analysis indicated Xinjiang Province as the most likely origin, although genomic surveillance for PPRV is poor and lack of sampling from other regions could bias this result. Times of most recent common ancestor (TMRCA) were June 2015 (95 per cent highest posterior density (HPD): August 2014 to March 2016) for all Mongolian PPRV genomes and May 2016 (95 per cent HPD: October 2015 to October 2016) for Mongolian wildlife-origin PPRV. This suggests that PPRV was circulating undetected in Mongolia for at least 6 months before the first reported outbreak in August 2016 and that wildlife were likely infected before livestock vaccination began in October 2016. Finally, genetic variation and positively selected sites were identified that might be related to PPRV emergence in Mongolian wildlife. This study is the first to sequence multiple PPRV genomes from a wildlife outbreak, across several host species. Additional full PPRV genomes and associated metadata from the livestock–wildlife interface are needed to enhance the power of molecular epidemiology, support PPRV eradication, and safeguard the health of the whole ungulate community.

## 1. Introduction

Peste des petits ruminants (PPR) is a contagious viral disease of sheep and goats with high morbidity and mortality rates, which is a major barrier to sustainable small ruminant production and dependent livelihoods and economies (FAO and OIE 2015; Jones et al., 2016). Consequently, PPR is the only livestock disease currently targeted by a Global Eradication Programme (GEP), which aims to rid the world of PPR by 2030 through vaccination of livestock and thereby contribute to achieving the Sustainable Development Goals. The aetiological agent, peste des petits ruminants virus (PPRV), has a broad host range, with serological or virological evidence of natural infection in a growing list of wild species within the order Artiodactyla (Munir 2014; Aziz Ul et al., 2018; Dou et al., 2020; Fine et al., 2020). PPRV infection of both captive and free-ranging wildlife may result in severe outbreaks and mortality, threatening species’ survival and ecosystem integrity. PPRV has caused mass mortality of mountain caprine species categorised as vulnerable by the International Union for Conservation of Nature (IUCN) (Michel and Ghoddousi 2020; Weinberg and Ambarli 2020), with >1,000 deaths of wild goats (*Capra aegagrus*) and sheep (*Ovis orientalis*) in Iran (Marashi et al., 2017) and >750 wild goats in Iraq (Hoffmann et al., 2012). Fatal PPR outbreaks have also been reported in free-ranging Sindh ibex (*Capra aegagrus blythi*) in Pakistan (Abubakar et al., 2011) and in ibex (*Capra ibex*) (Xia et al., 2016; Zhu et al., 2016; Li et al., 2017), bharal (*Pseudois nayaur*) (Bao et al., 2011, 2012; Li et al., 2017; Xia et al., 2016; Zhu et al., 2016), argali sheep (*Ovis ammon*) (Li et al., 2017), goitered gazelle (*Gazella subgutturosa*) (Li et al., 2017), and Przewalski’s gazelle (*Procapra przewalskii*) (Li et al., 2019) in China. To date, the most devastating impact of PPRV on biodiversity was its emergence in the critically endangered Mongolian saiga antelope (*Saiga tatarica mongolica*) in 2016–2017, which caused a mass mortality event and contributed to loss of ∼80 per cent of the population (Aguilar et al., 2018; Pruvot et al., 2020). In contrast, clinical disease has not been confirmed in free-ranging wildlife in Africa, despite high apparent PPRV seropositivity in wildlife populations in East Africa (Mahapatra et al., 2015; Fernandez Aguilar et al., 2020). The only published disease outbreak in free-ranging wildlife in Africa occurred in Dorcas gazelles (*Gazella dorcas*) in Dinder National Park, Sudan (Asil et al., 2019). However, this was not supported by field data to confirm the nature of the epidemic or event, and so whether this represents true wildlife disease remains equivocal, while African species in captivity have been shown to express PPR disease in zoological collections in the Middle East (Furley, Taylor, and Obi 1987; Kinne et al., 2010; Berkowitz et al., 2019). Therefore, while it is now clear that PPRV poses a threat to biodiversity, the determinants of differential disease expression among wildlife hosts are not understood. There are also significant knowledge gaps regarding the role of wildlife in the epidemiology and evolution of PPRV. It remains unclear whether wildlife can maintain or transmit the virus to livestock and thereby pose a threat to the PPR GEP.

It is important to assess the genetic diversity of PPRV to understand whether host range plasticity and viral virulence are linked to genetic changes in the virus. PPRV is a morbillivirus with a negative sense single-stranded RNA genome of ∼16 kilobases, which encodes six structural proteins, the nucleocapsid (N), phosphoprotein (P), matrix (M), fusion (F), haemagglutinin (H), and polymerase (L) proteins, and two non-structural proteins, V and C. The infectivity of PPRV is mediated by its envelope glycoproteins, H and F, which are therefore key viral determinants of cellular and host tropism. H binds the morbillivirus receptors SLAM and nectin-4 on immune and epithelial host cells, respectively, while F mediates the subsequent membrane fusion events to enable cell entry. The efficiency of receptor usage and entry into target cells are likely to be critical barriers to the emergence of morbilliviruses in atypical hosts. A recent study showed that a single amino acid substitution in PPRV H enabled it to use human SLAM as an entry receptor (Abdullah et al., 2018). Studies on the related morbillivirus canine distemper virus have also shown that only one or two amino acid changes in H are associated with host range expansion in nature (Ohishi et al., 2014; Nikolin et al., 2017) or via *in vitro* adaptation (Bieringer et al., 2013). The crystal structure of measles virus (MeV) H protein in complex with marmoset SLAM has been solved (Hashiguchi et al., 2011) and shows that the receptor-binding domain (RBD) comprises four sites on MeV H which interact with SLAM and which are well conserved in PPRV H (Abdullah et al., 2018). Several recent mutagenesis studies have also identified amino acid residues in PPRV H important for its ability to bind SLAM (Meng et al., 2020) and induce cell fusion (Gallo et al., 2021). In addition to cell entry, PPRV evidently requires efficient replicative and immune-evasive abilities for successful infection of atypical hosts, but the role of other viral genes in host range remains obscure.

PPRV is classified into four genetically distinct lineages, which can be discriminated based on phylogenetic analysis of short gene regions, often a few hundred nucleotides of the N gene (Couacy-Hymann et al., 2002; Kumar et al., 2014). Lineage IV viruses have dominated both the host range and geographic expansion of PPRV seen in recent years and are now replacing other lineages in many African countries (Libeau, Diallo, and Parida 2014; Tounkara et al., 2018, 2021; Dundon, Diallo, and Cattoli 2020). Understanding this expansion is critical to mitigate challenges to the PPR GEP and to understand the threat of PPRV to biodiversity. To do so necessitates the phylogenetic resolution provided by full genome sequencing using high coverage high throughput sequencing technologies (Baron et al., 2017), which is particularly important since such limited molecular epidemiological data on PPRV in wildlife exist at the global level. Earlier molecular evolutionary studies of PPRV based on full genomes have included a few wildlife-origin sequences (Muniraju et al., 2014; Shabbir, Rahman, and Munir 2020b). However, no studies have hitherto used phylogenomic approaches to address inter-species transmission patterns of PPRV.

In Mongolia, PPR was first confirmed in August 2016 (https://wahis.oie.int/#/report-info?reportId=8043, last accessed 23 June 2021), and a full PPRV genome was generated from livestock sampled in September 2016 (Shatar et al., 2017). The outbreak in Mongolian wildlife was laboratory-confirmed in December 2016 and led to mortalities of Mongolian saiga antelope (*Saiga tatarica mongolica*), goitered gazelle (*Gazella subgutturosa*), Siberian ibex (*Capra ibex sibirica*), and Argali (*Ovis ammon*), thought to have been caused by spillover of the virus from livestock and subsequent spread among wild ungulates https://wahis.oie.int/#/report-info?reportId=10463, (last accessed 23 June 2021); (Pruvot et al., 2020). Previously, the only molecular data for PPRV from Mongolian wildlife were partial N gene sequences from two saiga antelope (Pruvot et al., 2020). Here, we generated five novel full genome sequences for the PPRV which emerged in three species of Mongolian wildlife: saiga antelope, goitered gazelle, and Siberian ibex. Using these sequences and all other PPRV genomes available in GenBank from both wildlife and livestock hosts, we performed phylogenetic and molecular evolutionary analyses to address PPRV emergence in Mongolian wildlife and dynamics at the livestock–wildlife interface.

## 2. Results

### 2.1 Tissue distribution of PPRV in Mongolian wildlife

Total RNA was extracted from tissue samples collected at necropsy from four saiga antelope, one goitered gazelle and one Siberian ibex (Table S1). To determine the tissue distribution of PPRV replication in the wildlife hosts, reverse transcription polymerase chain reaction (RT-PCR) for a 350-nucleotide region of the N gene was performed on all available samples. Every tissue tested was RT-PCR-positive (Fig. S1), with an amplicon of the expected size, namely liver and ocular swab from Siberian ibex; tongue, soft palate, and lung samples from goitered gazelle; and tongue, soft palate, ocular and nasal swabs, gum scurf, mesenteric lymph node, spleen, liver, lung, heart, and blood from saiga antelope. Nucleic acid sequencing showed that this N gene region was identical in all six wildlife hosts and to the two published partial N gene sequences from saiga (Pruvot et al., 2020) and differed from the Mongolian livestock PPRV (KY888168.1) by two nucleotides (data not shown).

### 2.2 PPRV genome sequences from Mongolian wildlife hosts

Using the Illumina NextSeq sequencing platform, five new PPRV genomes were obtained for three wildlife species: three individuals of the Mongolian saiga antelope, one goitered gazelle, and one Siberian ibex (Table S2). The genomes were 15,954 nucleotides in length and contained a six-nucleotide insertion within the 5ʹ untranslated region (UTR) of F gene (at position 5216 in the alignment), shared by PPRV from Mongolian livestock (KY888168.1) and Chinese lineage IV strains after 2013 (Bao et al., 2017), but not observed in other published PPRV genomes. Two of the PPRV genomes from saiga had complete nucleotide coverage across the entire genome (saiga_3 and saiga_4), whereas another sequence from saiga (saiga_1) contained sequence gaps totalling 1133 nucleotides, the goitered gazelle sequence had a 33-nucleotide gap (in the M–F intergenic region), and the ibex sequence a 759-nucleotide gap (in the M–F intergenic region, plus 39 nucleotides of the F gene, which was later confirmed by F gene RT-PCR). The two complete PPRV sequences from different saiga individuals differed from each other at only three nucleotide sites. Aligning the PPRV sequences from saiga with the only full PPRV genome available for Mongolian livestock (KY888168.1), showed 99.7 per cent nucleotide identity, i.e. out of 15,954 nucleotides in the genome, there were 42 (saiga_4) or 45 (saiga_3) nucleotide differences.

### 2.3 Evolutionary rates and lineage divergence of PPRV

Following sequence curation as described below, seventy-six PPRV genomes from Genbank were added to the five novel PPRV genomes generated in this study, yielding a total of eighty-one sequences for phylogenetic analysis. These spanned 49 years from 1969 to December 2018 and included isolates from twenty-four countries. Previous phylogenomic analyses have included sequences found to be unreliable by our recombination analysis (Muniraju et al., 2014; Bao et al., 2017; Clarke et al., 2017; Shabbir, Rahman, and Munir 2020b), which we excluded (see methods section and Table S3). We therefore first analysed the evolutionary rates and global lineage diversification of PPRV using our Bayesian time-scaled phylogeny of eighty-one genomes.

**Figure 1.**
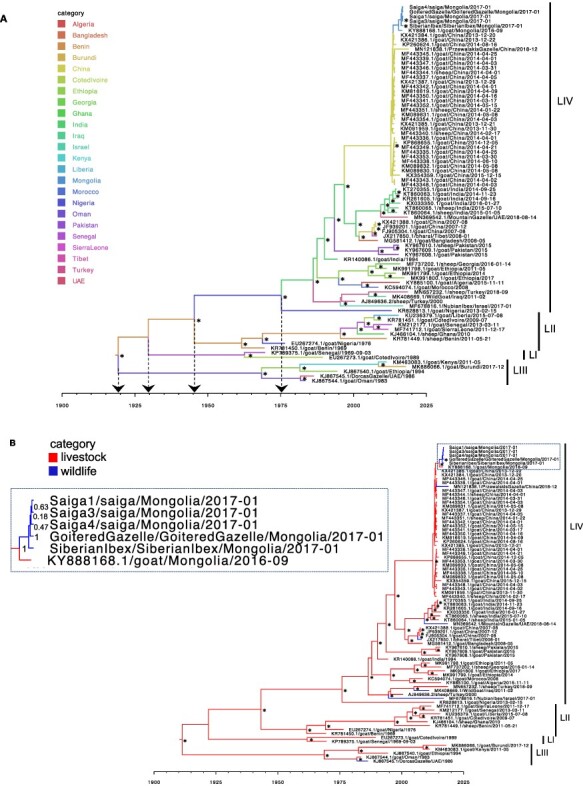
Bayesian time-scaled MCC trees using country (A) or host category (B) partitions. Bayesian phylogenetic analysis (*n* = 81 genomes) was run using BEAST v1.10.4, and trees are the combined output of three (A) or two (B) independent MCMC chains, visualized in FigTree. x-axis shows date. Branches are colour-coded by country (A) or host (B) as inferred using discrete trait analyses. Lineages, referred to as LI, LII, LIII, or LIV, are shown. Arrows to the x-axis in (A) show ancestral nodes and corresponding TMRCAs for different lineages. *Posterior probability >0.9 at the node opposite. A panel (demarcated by the dashed box) in Fig. 1B shows an enlargement of the Mongolian PPRV clade, with posterior probabilities shown opposite the corresponding nodes.

The mean evolutionary rate across the phylogeny, under an uncorrelated relaxed clock model found to be the best fit for the data, was 9.22E-4-nucleotide substitutions/site/year (95 per cent HPD interval: 6.78E-4–1.17E-3) (Fig. 1A). Table 1 gives the countries of origins and divergence times of PPRV lineages inferred from the Bayesian phylogenetic analysis. For lineage IV, which is expanding its geographic and host range, the median TMRCA was estimated to be 1975 (95 per cent HPD: 1961–1985) and its country of origin was inferred as Nigeria, with moderate support (67 per cent root state posterior probability).

**Table 1.** Time to the most recent common ancestor (TMRCA) and country of origin of PPRV lineages

PPRV lineage	TMRCA		Country of origin	
	Median	95% HPD interval	Country	Root state posterior probability (%)
All (root)	1919	1884–1945	Benin	46.0
I	1930	1902–1949	Benin	52.4
II	1945	1926–1959	Benin	55.5
III	1919	1884–1945	Benin	46.0
IV	1975	1961–1985	Nigeria	67.0

TMRCA and countries of origin were inferred from the Bayesian full genome phylogeny shown in Fig. 1A using FigTree. TMRCA is given to the nearest complete year. Note that country of origin assignment is limited to only those countries represented in Fig. 1A.

### 2.4 Phylogenetic analysis of PPRV at the livestock–wildlife interface

Including the five novel genomes from this study, twelve wildlife-origin PPRV genomes are currently available (Table S4), although one of these was excluded from our phylogenetic analysis (KT633939.1/ibex/China/20 January 2015). Two cases occurred in zoological collections, and the other ten PPRV genomes were from infections of free-ranging wildlife. Eleven of the wildlife PPRV genomes belong to lineage IV and one to lineage III. The only countries having both wildlife and livestock sequences were Mongolia and China. In contrast, three Middle Eastern countries, Israel, Iraq and UAE, had full PPRV genomes available in GenBank from wildlife hosts, but no genomes from infected livestock were available for these countries. Table S4 summarises the key epidemiological data for the 2016–2017 outbreak in Mongolian wildlife (Kock 2017; Pruvot et al., 2020) and the disease events associated with the other available wildlife-origin PPRV genomes.

Assessing the host-traited maximum clade credibility (MCC) tree shows that the wildlife-origin PPRV from China after 2013, i.e. MN121838.1 (Przewalski’s gazelle), lies within the clade of livestock PPRV (Fig. 1B). In contrast, several other wildlife PPRV genomes lie on branches that are basal to clades circulating in nearby locations. For example, PPRV from a bharal (sampled in Tibet in 2008) was basal to Chinese livestock sequences from 2007, PPRV from a mountain gazelle (sampled in UAE in 2018) was basal to a clade of six livestock sequences from India in 2014–2016, and PPRV from a Nubian ibex (sampled in Israel in 2017) was on a long branch basal to isolates from Turkey and Iraqi Kurdistan close to the Turkish border (Fig. 1B). Similar phylogenetic relationships for wildlife-origin PPRV genomes were seen using maximum likelihood (ML) phylogenetic reconstruction (Fig. S2). The occurrence of wildlife sequences as isolated sequences within the broader livestock diversity is consistent with repeated dead-end spillovers of PPRV from livestock into wildlife, but the lack of sequences from wildlife, long branches, and geographic distances between the most related livestock and wildlife sequences makes it impossible to rule out the transmission from largely undetected outbreaks in wildlife into livestock.

### 2.5 Phylogenetic analysis and TMRCA for PPRV emergence in Mongolian wildlife

The five novel genomes from wild Mongolian ungulates were most closely related to PPRV from a Mongolian goat (KY888168.1) sampled in September 2016, the only PPRV genome sequence available from Mongolian livestock. There was strong support for monophyletic grouping of the Mongolian wildlife and livestock sequences using both Bayesian (Fig. 1A, B) and ML (Fig. S2) inference methods. There was also strong support for the grouping of all five Mongolian wildlife sequences and, although poor support for the clade structure within the Mongolian wildlife clade, in every analysis the Siberian ibex formed a sister branch to the four other wildlife sequences from saiga and the goitered gazelle (Fig. 1A, B, Figs S2 and S3). The Mongolian sequences lie within a strongly supported clade of lineage IV sequences from China, which includes livestock sequences from 2013 to 2015, and a Przewalski’s gazelle sequence from 2018.

The dates of PPRV emergence in Mongolia and its wildlife were inferred using TMRCA analysis of the Bayesian time-scaled MCC tree shown in Fig. 1A. The median TMRCA of the six Mongolian PPRV genomes was June 2015 (95 per cent HPD: August 2014–March 2016). The median date for the MRCA for the five Mongolian wildlife PPRV sequences was May 2016 (95 per cent HPD: October 2015–October 2016). The ancestor at the node linking all the Mongolian PPRV sequences with the most closely related Chinese sequences was dated to July 2013 (95 per cent HPD: March 2013–November 2013). To check that the data partitioning for the traited analysis did not substantially alter the TMCRA analysis, an untraited phylogeny was also analysed (Fig. S3), which gave very similar results.

### 2.6 Phylogeographic analysis of PPRV emergence in Mongolian wildlife

SpreaD3 was used to visualise the geographic spread inferred through discrete phylogeographic analysis and identify well-supported rates using Bayes factor tests. This found that PPRV spread from China into Mongolia was very strongly supported with a Bayes factor of 494 and associated posterior probability of 0.96 (Table S5).

To further explore and visualise phylogeographic patterns in the data, samples were geocoded and analysed using Microreact (www.microreact.org). Samples were geocoded at the highest resolution possible. Global Positioning System (GPS) sampling locations were available for all Mongolian wildlife samples (Table S1), the Mongolian livestock sample, and the 2008 bharal sequence from Tibet. Of the other thirty-three PPRV genomes within the Chinese 2013–2018 clade, twenty-eight samples had province-level location data, hence region centroids were used for geocoding, while country-level location only was available for five sequences, in which case the China centroid was used. An open-access interactive dynamic visualisation of our global PPRV data set, integrating phylogenetic, spatial, temporal, and host (wildlife/livestock host) data, is available at the permanent link https://microreact.org/project/5WNeX14MRFvwe8YLhn5a1S/e2d5dafd (last accessed 23 June 2021, to be updated as further genomes become publicly available).

**Figure 2.**
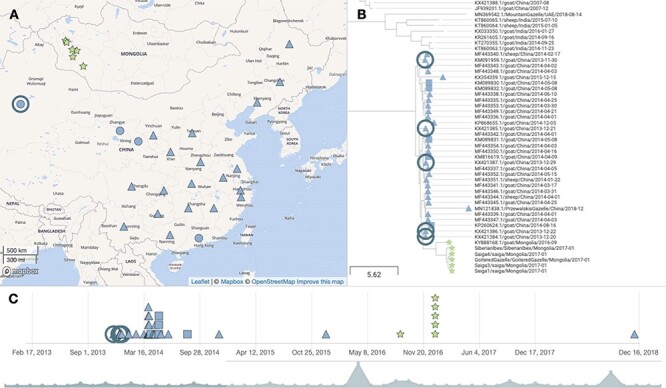
Phylogeographic visualization. An MCC phylogenetic tree (81 sequences) was uploaded to Microreact together with geocoded locations of PPRV genomes and metadata to produce the dynamic visualization of phylogenetic, spatial, and temporal relationships of the global PPRV data set. The figure shows one view available at the Microreact project link (https://microreact.org/project/5WNeX14MRFvwe8YLhn5a1S/e2d5dafd), showing the location (A), phylogenetic relationship (B), and timeline (C) for the clade of PPRV genomes from Mongolia and China since 2013. Symbol colour denotes the country of origin (Mongolia in green and China in blue). The symbol shape denotes different resolutions of geocoding for genomes: stars denotes samples with GPS coordinates; triangles denote region centroids; squares denote country centroids, and a circle on the map view indicates multiple genomes from the same location. The ringed samples in panels (A)–(C) show samples from Xinjiang province, including KX421386.1 and KX421384.1. The map shown in (A) uses base map and data from OpenStreetMap and OpenStreetMap Foundation, available under the Open Database License, with tiles from the Mapbox mapping platform, used via the freely available Microreact application.

The map highlights the proximity of the sampling locations for the wildlife and livestock PPRV genomes in the Western Mongolian provinces of Khovd and Gobi Altai, with <40 km between the livestock sample and one of the saiga antelope sampled in Khovd near the Khar-Us lake 4 months later (Fig. 2). The Siberian ibex sample, from Tugrug soum of Gobi-Altai (Table S1), was furthest from the sampling location of the Mongolian livestock (∼355 km) and marginally closer to the Chinese border than other detected cases.

The PPRV genomes KX421386.1 and KX421384.1, from December 2013, are closely phylogenetically related to the Mongolian sequences and also the geographically closest sequences, from Xinjiang province of Northwest China, which borders Mongolia (Fig. 2).

### 2.7 Amino acid polymorphisms associated with host range expansion

The five new PPRV sequences were compared with the seventy-six other genomes in our data set in order to identify polymorphisms of interest that might be associated with PPRV emergence in Mongolian wildlife (Table 2). All point mutations identified by next-generation sequencing (NGS) in the H and F genes of the Mongolian wildlife-origin PPRV were confirmed by direct H and F gene RT-PCR and Sanger sequencing.

**Table 2.** Amino acid polymorphisms

		Residue present					
		Mongolian wildlife species					
PPRV gene	AA position	Saiga	Ibex	Gazelle	Mongolian livestock (KY888168.1)	China clade since 2013	Other PPRV genomes
H	*112*	S	**G**	S	S	S	S in all
	*157*	**R**	**R**	**R**	**R**	K	K in all
	*244*	**A**	**A**	**A**	**A**	T	T in all
	*263*	L	L	L	**F**	L	F in LIII; L in all other genomes
	*315*	**R**	**R**	**R**	**R**	**R**	K in all
	*450*	**K**	**K**	**K**	**K**	**K**	G in two LIV sequences; S in two LI sequences; R in all others
	*506*	D	D	D	**N**	D	D in all
	*546*	**S**	**S**	**S**	**S**	**S**	G in all
F	*66*	K	K	K	**R**	K	R in MG581412.1 (LIV); T in KR828813.1 (LIV); all others K
	*518*	K	K	**N**	K	K	K in LIV; R in LI, LII, & LIII (except K in LII EU267274.1)
P	*137*	**A**	**A**	**A**	**A**	V	V is the consensus; a few sequences encode L, F, or I
	*285*	**P**	**P**	**P**	**P**	S	S in other LIV from Asia; L in LIV from Africa and Middle East & LI, LII, & LIII
	*509*	**L**	**L**	**L**	**L**	P	P in all; except L in two Middle Eastern LIV sequences
V	*137*	**A**	**A**	**A**	**A**	V	V is the consensus; a few sequences encode L, F, or I
	*285*	**A**	**A**	**A**	**A**	V	V in all
N	*484*	N	N	N	**K**	N	T in four sequences (from LI & LIII); N in all other

The amino acid sequences for each PPRV gene were compared between Mongolian, closely related Chinese, and other PPRV genomes to identify polymorphic sites. The amino acid positions and encoded residues for identified polymorphic sites are shown. Under ‘Other PPRV genomes’, lineages are referred to as LI, LII, LIII, or LIV. Bold letters indicate amino acid residues of interest occurring in Mongolian PPRV (either livestock or wildlife origin).

Among the polymorphisms observed, the Mongolian livestock PPRV (KY888168.1) encodes asparagine (N) at position 506 of PPRV H, whereas every other sequence encodes aspartate (D) as part of the completely conserved ^505^DDD^507^ motif. The PPRV H from Siberian ibex has a glycine (G) at amino acid 112, whereas the other eighty sequences in the data set encode serine (S). In addition, two residues in H are unique to the Mongolian PPRV sequences, including the five wildlife and one livestock sequence: arginine (R) instead of lysine (K) at amino acid 157, and hydrophobic alanine (A) instead of hydrophilic threonine (T) at position 244. At several sites within the H gene, the monophyletic clade of thirty-nine Chinese/Mongolian sequences encodes common signature residues compared to other PPRV sequences (R315, K450, and S546) (Table 2). For the F protein, PPRV from goitered gazelle was the only genome to encode asparagine (N) at position 518, a significantly different residue from lysine (K) (in all other lineage IV genomes) or R (lineages I, II, and III). All Mongolian PPRV sequences also encode unique residues in their P proteins (A137 and P285) and V proteins (A137 and A285) (Table 2).

### 2.8 Molecular modelling of polymorphic amino acids in Mongolian PPRV H

**Figure 3.**
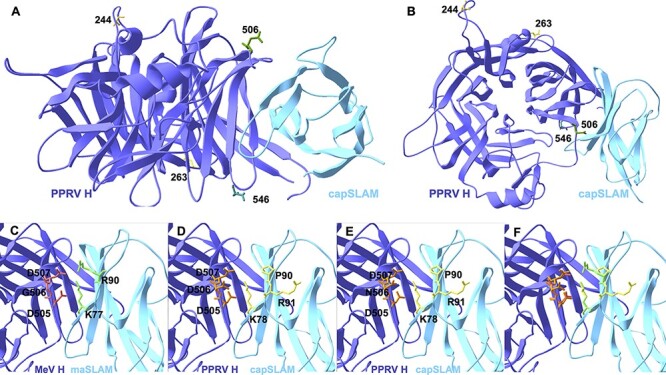
Homology modelling of the PPRV H-SLAM complex. Modelling was performed using SWISS_MODEL using the published crystal structure of MeV H bound to marmoset SLAM to show positions of Mongolian-specific amino acid polymorphisms in PPRV H. Side (A) and top (B) orthogonal views of PPRV H bound to caprine SLAM (capSLAM) showing residues for PPRV H from saiga antelope (A244, L263, D506, S546). Residues at site 1 of the RBD for (C) MeV H and marmoset SLAM (maSLAM), (D) PPRV H D506 and capSLAM, (E) PPRV H N506 and capSLAM, or (F) overlayed image of panels C and D. H proteins are shown in purple and SLAM proteins in turquoise.

To determine the location of the polymorphic sites identified in PPRV H from Mongolian wildlife and livestock, structural homology modelling was performed, based on the solved crystal structure of the head domain of MeV H in complex with SLAM, the host cell entry receptor for morbilliviruses (Hashiguchi et al., 2011). Of the polymorphic sites identified between H sequences, sites 112 and 157 were not captured by the crystal structure. Amino acid residues 244 and 263 were predicted to be distant from the SLAM-binding interface and surface exposed (Fig. 3A, B). Residues 506 and 546 were predicted to lie within the region that forms the SLAM binding interface (Fig. 3A, B). While amino acid site 546 of H is not thought to form a direct contact with SLAM, amino acid 506 lies between two residues (D505 and D507), which in MeV H form salt bridges to K77 and R90 of marmoset SLAM, comprising site 1 of the RBD (Fig. 3C) (Hashiguchi et al., 2011). Modelling caprine SLAM in place of marmoset SLAM reveals K78 in place of K77, whereas R90 in marmoset SLAM is replaced by R91 in caprine SLAM, with the preceding P90 facing away from PPRV H (Fig. 3D). Replacing G506 of MeV H with D506 (i.e. the consensus residue in PPRV H) or N506 (i.e. the substitution seen in PPRV from Mongolian livestock) could affect the interaction with SLAM due to its proximity (Fig. 3C–F).

### 2.9 Selection pressure analysis

To test for positive selection, methods were used that assess the numbers of non-synonymous (dN) to synonymous (dS) nucleotide substitutions per site, with dN > dS indicative of positive selection (i.e. adaptive evolution). To assess positive selection acting on individual codons, the coding sequence (CDS) of each PPRV gene, except P, V, and C which are expressed from overlapping reading frames, was analysed using Mixed Effects Model of Evolution (MEME), Fast Unconstrained Bayesian Approximation (FUBAR), Fixed Effects Likelihood (FEL), and CodeML. Sites under positive selection were identified in all analysed genes with all four methods, except for the F gene (three methods) and the M gene (one method) (Table 3, Table S6). The amino acid positions identified as evolving under positive selection by all four methods were amino acid 246 of the H protein and amino acid 616 of the L protein.

**Table 3.** Amino acid sites under positive selection

Protein	MEME	FUBAR	FEL	CodeML
N	*46, 431*	*456*	*211, 431, 467*	*424, 456*
M	*311*	–	–	–
F	*3, 8, 11*, *46, 145, 368, 530*	*8*	*8*	–
H	*21, 210, 211, 245, 246, 288, 305*, *330, 339, 476, 574*	*246*	*246, 476, 574*	*246*
L	*54, 68, 113, 124, 230, 336, 349, 614*, *616, 719, 720, 899, 1343, 1708, 1901, 2038, 2080, 2115*	*614*, *616, 1257*	*614, 616, 623, 1257*	*616*

Each indicated PPRV gene was tested for site-specific selection across the phylogeny using the four analysis methods shown: MEME, FUBAR, FEL, and CodeML. Amino acid positions identified as evolving under positive selection by each method are shown, using their default threshold of significance (*P* ≤ 0.05 for CodeML, *P* ≤ 0.1 for MEME, FUBAR, and FEL). Sites with significance 0.05 ≤ *P* < 0.1 are shown in italics. ‘–’ indicates no positively selected sites were identified for that gene using that method.

Methods for detecting lineage-specific selection were also used to test whether the clade of Mongolian and Chinese PPRV sequences since 2013 showed evidence of positive selection. Both Branch-site Unrestricted Statistical Test for Episodic Diversification (BUSTED) and adaptive Branch-Site Random Effects Likelihood (aBSREL) found no evidence of selection for N, M, F, or H genes but did find evidence of positive selection acting on the L gene of the Mongolian/Chinese PPRV clade (*P* < 0.05) (Table 4, Table S7). The BUSTED result indicates L gene-wide episodic diversifying selection, i.e. evidence that at least one site on at least one test branch within the clade has experienced diversifying selection. aBSREL identified episodic diversifying selection acting on two branches, namely the single Mongolian livestock PPRV sequence (KY888168.1) and the PPRV L gene from Chinese goat (KP260624) (Table S7). FEL identified positively selected sites within each gene in the Chinese/Mongolian clade (Table 4). Some sites were identified both by lineage-specific FEL and earlier by MEME (Tables 3 and 4).

**Table 4.** Lineage-specific selection tests

Protein	BUSTED	FEL	aBSREL
N	–	**46**, *423, 426, 484*	–
M	–	**311**	–
F	–	*187*, *518*, **530**	–
H	–	**21**, 112, 162, *244*, **305**, **330**, **339**, 394, 410, 438, 440, *506*, 575, 590	–
L	+ (LRT = 18.64; p= 4.5 × 10^−5^)	**68**, *87*, **113**, 121, **124**, *152,* 277, 299, **336**, 485, 617, 647, 707, ***719***, **720**, 7,231,022, 1031, 1198, 1264, 1272, **1343**, *1375*, 1452, *1622*, 1656, *1715*, **1901**, 1990, 2010, **2038**, 2089	+ (see Table S7)

The clade of China/Mongolia sequences was selected as foreground branches on which to test for positive selection using the three analysis methods shown: BUSTED, FEL, and aBSREL. ‘–’denotes no evidence of positive selection, as assessed using LRT using the default threshold of significance for BUSTED and aBSREL (*P* ≤ 0.05). For FEL, amino acid sites identified as under positive selection are indicated (using the default threshold of significance of *P* ≤ 0.1). Sites with significance 0.05 ≤ *P* < 0.1 are shown in italics. Sites in bold were also identified by MEME in phylogeny-wide testing.

Eight sites at which Mongolian PPRV-specific amino acid polymorphisms had been noted in either wildlife or livestock (Table 2) were also identified as positively selected by the lineage-specific FEL analysis (Table 4), suggesting the functional significance of these sites, namely H_112, H_244, H_506, F_518, and N_484.

## 3. Discussion

Despite the broad host tropism and impact of PPRV across wild ungulate species, there is a paucity of wildlife-origin PPRV genomes, which, along with the lack of field epidemiological data, has hindered the understanding of viral evolution and dynamics at the wildlife–livestock interface. This understanding is critical in order to design effective disease control and eradication strategies and thereby support the success of the PPR GEP and mitigate the threat of PPRV to both domestic and wild ungulates. In this study, full PPRV genomes were generated from three Mongolian saiga antelope, one goitered gazelle, and one Siberian ibex that were part of a major mortality event in Mongolian wildlife in 2016–2017. These were analysed together with a curated set of all other genomes available in GenBank, to examine PPRV evolution and cross-species transmission. This showed strong support for monophyletic grouping of genomes from Mongolian wildlife and livestock and for incursion of PPRV into Mongolian from China. Our TMRCA analysis also indicated that PPRV emerged in Mongolia’s endangered wildlife populations before livestock vaccination commenced, and sequence polymorphisms and signatures of positive selection were identified.

Prior to our phylogenetic analysis, recombination detection algorithms were applied to our data set and identified several potential recombinant PPRV genomes. Recombination has never been reported for PPRV and is rare among negative-sense RNA viruses (Han and Worobey 2011; Beaty and Lee 2016). However, bioinformatics errors or laboratory contamination can lead to false signals of recombination in PPRV (Shabbir, Rahman, and Munir 2020a,b). Previous reports have also highlighted the issue of contamination in published PPRV sequences (Liu 2018; Dundon, Diallo, and Cattoli 2020; Shabbir, Rahman, and Munir 2020a,b), so the most probable explanation is that the recombinant PPRV genomes we identified are the result of laboratory contamination during the process of genome sequencing. However, PPRV recombination cannot be totally ruled out and should be further explored in dedicated infection experiments. The inclusion of dubious and/or recombinant genomes in earlier phylogenomic studies may have influenced their results (Muniraju et al., 2014; Bao et al., 2017; Clarke et al., 2017; Shabbir, Rahman, and Munir 2020b), and we therefore first analysed PPRV molecular evolution globally.

The genome-wide mean evolutionary rate inferred from the Bayesian MCC phylogeny of eighty-one PPRV genomes was 9.22E-4-nucleotide substitutions/site/year (95 per cent HPD interval: 6.78E-4–1.17E-3), which is comparable to previous estimates (Muniraju et al., 2014; Adombi et al., 2017; Shabbir, Rahman, and Munir 2020b). The TMRCA and country of origin of each of the PPRV lineages were inferred from our Bayesian MCC phylogeny (Table 1). In the case of lineage IV, which is becoming the predominant lineage globally, the median TMRCA was estimated as 1975 (95 per cent HPD: 1961–1985). This date is slightly earlier than that reported from the analysis of more limited data sets of twelve (Muniraju et al., 2014) or twenty-seven genomes (Adombi et al., 2017), although the HPD intervals overlap, or from partial N or F gene data sets (Muniraju et al., 2014; Padhi and Ma 2014), and broadly equivalent to estimates obtained from the analysis of fifty-three full genomes (1968) (Clarke et al., 2017).

All phylogeographic analyses are affected by sampling bias. Likewise, our analysis can only ascribe countries of origin for PPRV lineages to one of the countries represented by a genome sequence in our data set. Our analysis indicates with moderate support that the country of origin of lineage IV was Nigeria, with the second most supported origin in Benin. Previous analyses identified India as the most likely origin of lineage IV, but recent sequences from Nigeria were not included in that analysis (Muniraju et al., 2014). The extremely poor sampling prior to the 1990s means that while an origin of lineage IV in West Africa seems plausible, the lack of genomic samples from other countries in this region precludes us from robustly establishing the country of origin. However, the detection of lineage IV PPRV in livestock in Cameroon in 1997, in the Central African Republic in 2004, and in several other Northern and Eastern African countries in the early 2000s, in the absence of known animal movement from Asia (Banyard et al., 2010; Kwiatek et al., 2011; Libeau, Diallo, and Parida 2014; Dundon, Diallo, and Cattoli 2020), also supports the hypothesis that lineage IV may have emerged in Africa before spreading to the Middle East and Asia and then re-emerging in African countries. While historical animal movement data are limited, the live animal trade from the Horn of Africa into Middle Eastern countries (Bouslikhane 2015) provides a plausible route for PPRV lineage IV spread out of Africa, and recent outbreaks also indicate transmission pathways linking North African to Eurasian countries (Donduashvili et al., 2018). Additional full genomes for lineage IV viruses from Africa, both historical and contemporary, would improve the phylogenetic power for more robust testing of this hypothesis.

Phylogenetic relationships between wildlife and livestock origin PPRV were assessed across the global phylogeny. With the exception of the Mongolian wildlife clade, all wildlife sequences occur as isolated sequences within the broader livestock diversity. For China, the only other country for which both wildlife and livestock genomes are available, phylogenetic analysis showed PPRV from Przewalski’s gazelle embedded, albeit with poor node resolution, within a well-supported clade of thirty-two livestock viruses sampled since 2013. No additional PPRV genomes were available from UAE, Iraq, or Israel, although eighteen full genomes from livestock in Israel between 1997 and 2004 were sequenced previously but unfortunately not made publicly available (Clarke et al., 2017). A more dense sampling of PPRV genomes globally would likely correct sampling bias and help reveal that viruses from wild and domestic hosts cluster together according to geographic location, as seen for China and Mongolia (Fig. 1 and Fig. S2), and indicated by partial N gene analysis (Rahman, Munir, and Shabbir 2019).

The novel PPRV genomes from Mongolian wildlife were most closely phylogenetically related to the PPRV genome from Mongolian livestock, strongly supported in both Bayesian (Fig. 1 and Fig. S3) and ML (Fig. S2) phylogenies. Although this might be expected, it was not evident in the earlier analysis, which showed closer phylogenetic proximity of saiga-origin PPRV to Chinese livestock as opposed to Mongolian livestock sequences, owing to an insufficient phylogenetic resolution of partial N gene sequence data used (255 nucleotides) (Pruvot et al., 2020). Our study is the first to sequence multiple PPRV genomes from an outbreak in wildlife. Taking advantage of this, the full PPRV genome available for Mongolian livestock (Shatar et al., 2017), and the dense sampling of PPRV genomes in China since 2013 (Bao et al., 2017), we examined PPRV emergence in Mongolian wildlife. The six Mongolian PPRV genomes formed a monophyletic group within a large clade (*n* = 39) that includes lineage IV PPRV from China sampled since 2013 (Fig. 1 and Fig. S2). In China, the first PPR outbreak was in Tibet in 2007–2008, and there were subsequently no reports of PPR until its re-emergence in Xinjiang province in November 2013 (Wu et al., 2016). PPRV from China in 2007/2008 (*n* = 4 genomes) was more closely phylogenetically related to viruses from India (spanning 2014–2016) than to the PPRV in China since 2013, consistent with earlier reports (Wu et al., 2016; Bao et al., 2017). Statistical testing of the Bayesian analysis of full genomes provided very strong phylogenetic support that PPRV spread into Mongolia from China (Bayes factor: 494; posterior probability: 0.96), although the lack of surveillance or genomic sequencing from other neighbouring countries (e.g. Kazakhstan and Russia) means that we cannot exclude introduction via an intermediary location. Phylogeographic visualisation using Microreact showed that PPRV genomes closely related to Mongolian PPRV were from livestock in Xinjiang province. Incursion of PPRV from Xinjiang province would be consistent with epidemiological reports that initial livestock cases and the earliest suspected wildlife cases clustered at the southwestern Mongolia–China border (Kock 2017; Pruvot et al., 2020). Between 2013 and 2016, PPRV cases in Argali sheep, *Capra ibex*, and goitered gazelle have been confirmed across six different counties within Xinjiang province, where shared grazing provides an opportunity for cross-species transmission (Zhu et al., 2016; Li et al., 2017). Owing to the presence of multiple PPRV-susceptible wild ungulate species in Xinjiang (Gao et al., 2011) and its extensive national borders (with Mongolia, Kazakhstan, Kyrgyzstan, India, Pakistan, Russia, Afghanistan and Tajikistan), Xinjiang should be a focus for increased surveillance and sampling of PPRV across the wild and domestic ungulate community.

The TMRCA of the clade of Mongolian PPRV genomes (one livestock and five wildlife) in our analysis was June 2015 (95 per cent HPD: August 2014–March 2016). The TMRCA linking the Mongolian to the Chinese genomes was inferred as July 2013 (95 per cent HPD: March 2013–November 2013). The introduction of PPRV into Mongolia likely occurred between the TMRCA estimates for these two nodes. Therefore, the very latest date of emergence in the country that is consistent with the phylogenetic analysis is March 2016 (with 95 per cent probability). This suggests that PPRV was circulating undetected for a minimum of 6 months before the first reported PPR outbreak in Mongolia, which was reported in livestock in Khovd province in August 2016 (notified to the World Organisation for Animal Health (OIE) in September 2016, Notification report REF OIE 20934). The TMRCA analysis indicates a median date for PPRV emergence in Mongolian wildlife of May 2016 (95 per cent HPD: October 2015–October 2016). This suggests that PPRV infections in wildlife may have pre-dated the first confirmed wildlife case in late December 2016, as proposed earlier based on interview data and undiagnosed wildlife mortalities (Kock 2017; Pruvot et al., 2020). However, more genomic sequencing of PPRV detections in livestock during 2015–2016 in this region is required to confirm this, as it is possible that ‘missing’ livestock sequences would intersperse with the (currently monophyletic) wildlife clade, thereby changing this interpretation. Pruvot et al. reported that earliest suspected unconfirmed cases in Mongolian wildlife occurred in Siberian ibex in July/August 2016 (Pruvot et al., 2020). All wildlife PPRV genomes in our analysis date from January 2017 and the clade branching structure is poorly supported in our analyses, thus no inference can be made regarding the relative timing of emergence among different wildlife species. Interestingly, however, phylogenetic reconstruction consistently showed PPRV from Siberian ibex located on a sister branch to the saiga and goitered gazelle viruses (Fig. 1, Figs S2 and S3). If the phylogenetic separation observed correctly captures true patterns of ancestry, phylogenetic separation could be related to (1) some structuring of the virus between wild species, potentially related to a longer period of transmission and evolution in ibex consistent with earlier emergence in this species, and/or (2) the phylogenetic structure seen could be related to the geographical separation of the ibex sample, which was further southeast than the other sampling sites.

Based on epidemiological and ecological evidence, multiple spillover events from livestock to different wildlife populations during the Mongolian outbreak have been proposed (Pruvot et al., 2020). The lack of additional livestock-origin genomes means that this cannot currently be confirmed using phylogenetic approaches, although our data are compatible with the hypothesis of spillover from domestic into wild ungulates in Mongolia. Our TMRCA analyses suggest that transmission of PPRV at the livestock–wildlife interface occurred prior to, or at the latest contemporaneous with, the livestock vaccination campaign that began in October 2016. This could explain the emergence in wildlife despite the vaccination of ∼10.4 million small ruminant livestock in Mongolia’s Western provinces, without inferring inadequacies in vaccination coverage or seroconversion (Kock 2017).

At the time of the disease outbreak, it is clear that the PPRV infecting wildlife was not only virulent but already well-adapted for infection and transmission in these hosts, at least in saiga antelope. It is possible that mutations present in the wildlife- infective PPRV strains, or their ancestral viruses, contributed to this, and therefore genomic sequences were analysed for notable sequence features and signatures of adaptive evolution. The genomes from Mongolian wildlife, as well as from Mongolian livestock and all PPRV from China since 2013, have a six-nucleotide insertion in the 5ʹ UTR of the F gene, which renders the genome 15,954 nucleotides long, instead of 15,948 nucleotides in all other PPRV strains (Bao et al., 2017; Li et al., 2015; Shatar et al., 2017; Zhu et al., 2016). This insertion maintains the ‘rule of six’, i.e. that genome length is a multiple of six, which is necessary for efficient replication of members of the *Paramyxoviridae* family (Calain and Roux 1993). Interestingly, similar insertions have also been observed in MeV and rinderpest virus genomes (Bankamp et al., 2014; Ivancic-Jelecki et al., 2016; Gil et al., 2018; King et al., 2020). Since the PPRV-F 5ʹ UTR is known to enhance F gene translation (Chulakasian et al., 2013), experiments to address the functional consequences, and potential selective advantage, of the observed insertion in the Mongolian and Chinese PPRV should be prioritised.

The CDS of each PPRV gene was assessed for non-synonymous nucleotide changes and these were correlated with the results of selection pressure analyses using multiple methods. Positive selection in viral genomes indicates adaptive evolution in response to changing fitness or functional requirements (Frost, Magalis, and Kosakovsky Pond 2018; Spielman et al., 2019), including infection and replication in novel hosts and evasion of host innate and adaptive immune responses, and is most likely at interaction interfaces between viral and host cell molecules.

For the H and F envelope entry proteins, polymorphisms specific to Mongolian wildlife PPRV were present at amino acid 112 in H from Siberian ibex and at amino acid 518 in F from goitered gazelle, with both sites under lineage-specific positive selection (Tables 2 and 4). More PPRV genomes would clearly be needed to assess the possibility that these are host species-specific mutations. Of note, however, are two polymorphisms in H which are specific to the six PPRV genomes from Mongolian livestock and wildlife, namely R157 (instead of K157 in all seventy five other genomes) and A244 (instead of T244 in all seventy-five other genomes), raising the possibility that these substitutions may have helped PPRV spillover to wildlife. Whereas K157R is not a major change in physiochemical or steric properties of the amino acid, the change from the polar, uncharged amino acid threonine (T) to hydrophobic alanine (A) at position 244 of the Mongolian PPRV H protein could have more significant effects on inter-molecular interactions. The adjacent residues 234–243 form a predicted T cell epitope (Agrawal et al., 2020) and therefore one possibility is that T244A influences interactions with MHC I. This region of H is also subject to adaptive evolution: site 244 is detected as under positive selection in the China/Mongolia linage using FEL methods, site 245 is detected by MEME as under episodic positive selection, and site 246 is identified by all four methods in phylogeny-wide testing (Table 3) and was also reported previously as a positively selected site (Liang et al., 2016). The single genome from Mongolian livestock has two substitutions in H, L263F and D506N, which are not shared by the five wildlife-origin PPRV and should be assessed in other livestock samples. If consistently seen, D506N is particularly noteworthy since it lies at the SLAM binding interface (Fig. 3) and all other PPRV strains encode aspartate (D) at this site (Abdullah et al., 2018). Neighbouring residues D505 and D507 are completely conserved among morbilliviruses (Abdullah et al., 2018) and comprise site 1 of the RBD (Fig. 3), forming salt bridges to SLAM in the crys tal structure for MeV H (Hashiguchi et al., 2011) and in homology models for PPRV H (Liang et al., 2016; Dou et al., 2020). Therefore, this residue could be functionally important for infectivity and viral tropism. Substitutions already present in the Chinese lineage of PPRV since 2013 may also have contributed to the emergence in Mongolian wildlife, including unique residues at sites 315, 450, and 546 of the H protein. At position 546, all sequences from the Mongolia/China clade encode serine, as does MeV at the equivalent position, whereas every other PPRV strain in the phylogeny encodes glycine. This residue is located adjacent to hydrophobic site 4 of the RBD (Fig. 3) but is not thought to form a direct contact with SLAM (Hashiguchi et al., 2011).

In addition to its role in genome encapsidation and replication, the N protein was shown recently also to inhibit type I interferon (IFN) by binding IRF3 (Zhu et al., 2019). We identified residues 46 and 426 in N as positively selected, with both MEME and FEL (Tables 3 and 4), as well as a Mongolian livestock-specific residue and lineage-specific positive selection at site 484 (Tables 2 and 4). The L gene, although lacking any Mongolian-PPRV-specific polymorphisms, was the only gene identified as positively selected by all three methods when the China/Mongolia clade was tested (Table 4), suggesting this catalytic polymerase protein has undergone adaptive evolution in this clade. Functional studies including pseudotyping (to assess receptor usage), minigenome (to assess replication), immune reporter, and intermolecular binding assays are beyond the scope of the current study but should be planned to define the role of the mutations identified here in lineage IV PPRV expansion.

In summary, our study has provided insights into PPRV transmission at the livestock–wildlife interface. More PPRV full genomes are required in order to strengthen molecular epidemiological studies. The occurrence of cases in wildlife should serve as a trigger to initiate local surveillance/sampling in livestock, and the surveillance and opportunistic sampling for wildlife disease events should be increased. However, quantifying the direction and extent of pathogen transmission in multi-host systems is challenging, even in the case of extremely detailed longitudinal study systems with pathogen genomic and host life history data (Crispell et al., 2019). There is thus a need for other types of data, including interspecific contact rates and pathways, and enhanced epidemiological and serological data, to enable approaches that integrate these with genomics (Viana et al., 2016; Crispell et al., 2019; Bataille et al., 2021) and can lead to an improved understanding of interspecific PPRV transmission dynamics and the epidemiological roles of wildlife species.

## 4. Materials and methods

### 4.1 Sample collection

Samples from five wild Mongolian ungulates suspected of being infected with PPRV were all collected in January 2017 during an emergency field mission to urgently respond, assess, and advise the Mongolian Government through the National Emergency Committee. The mission involved the Food and Agriculture Organization of the United Nations (FAO) Crisis Management Centre-Animal Health, the Wildlife Conservation Society, the Royal Veterinary College (RVC), the Veterinary and Animal Breeding Agency, and the State Central Veterinary Laboratory (SCVL) (Kock 2017). Tissues were collected from fresh carcass necropsies from three saiga antelope, one goitered gazelle, and one Siberian ibex. GPS locations of sampled animals are provided in Table S1. Total RNA was extracted from tissues using a NucleoSpin RNA Virus mini kit (Macherey-Nagel, 740956) at SCVL, Ulaanbaatar. Samples were then imported to RVC under APHA and CITES import permits.

### 4.2 RT-PCR

One-step RT-PCR was performed on 2 µl total RNA using the SuperScript IV One-Step PCR kit (ThermoFisher, Catalog No. 12594025) either to amplify nucleotides 1232–1583 of the PPRV N gene using published primers NP3 and NP4 (Couacy-Hymann et al., 2002) or to amplify the full-length H gene (with primers 5ʹ-CTCCACGCTCCACCACAC-3ʹ and 5ʹ-CTCGGTGGCGACTCAAGG-3ʹ) or the full-length F gene (with primers 5ʹ- GCTATGCGGCCGCACCATGACGCGGGTCGCAATYTT-3ʹ and 5ʹ-GGTGAGGATCCCTACAGTGATCTTACGTACGAC-3ʹ).

### 4.3 Whole-genome NGS

Total RNA from tissues was converted to complementary DNA with SuperScript IV and sequencing libraries prepared with the Nextera XT DNA Library Preparation Kit (Illumina, FC-131-1024). Sequencing was performed using the Illumina NextSeq platform with 150-bp paired-end reads. Sequencing data were mapped to a reference PPRV genome using Burrows-Wheeler Alignment tool (BWA) (Li and Durbin 2010), and then consensus calling was performed using SAMtools (Li et al., 2009). This was corroborated by removing host reads and then undertaking *de novo* assembly with SPAdes v3.8.0 (Bankevich et al., 2012), and then both outputs were aligned to confirm PPRV genome sequences.

### 4.4 PPRV full genome data set curation and recombination analysis

In addition to the five novel PPRV genomes, all full PPRV genomes available in Genbank were downloaded (last accessed 22 November 2020). Vaccine sequences were removed from the data set, as well as one sequence noted in GenBank as multiply passaged in cell culture (MN369543.1). Sequence alignment was performed using multiple alignment using fast Fourier transform (MAFFT) (Katoh et al., 2002) in the Geneious software package, followed by manual editing (e.g. trimming insertions in genomes KM089831 and KM816619). TempEst was run to assess temporal signal in the data and evidenced clock-like evolution (Fig. S4) (Rambaut et al., 2016). The alignment of eighty-five PPRV genomes was then analysed using the Recombination Detection Program (RDP) version 4.101 (Martin et al., 2015), using seven different recombination detection methods (RDP, GENECONV, BootScan, MaxChi, Chimaera, SiScan, and 3Seq) and default settings. Signatures of recombination were detected in four sequences (KR828814.1/goat/Nigeria/2012-05-09, KJ867541.1/goat/Ethiopia/2010, KJ867543.1/goat/Uganda/2012 and KT633939.1/ibex/China/2015-01-20) that were identified by at least five of the seven different detection algorithms (*P* < 0.01) (Table S3). These are most likely the result of laboratory contamination (Han and Worobey 2011), and these sequences were considered unreliable and removed from our data set, leaving a total of eighty one PPRV genomes (alignment provided in S1 File).

### 4.5 Phylogenetic analysis

The general time reversible (GTR) nucleotide substitution model with gamma-distributed variable rates (G) and some invariable sites (+I) best fitted our data set, according to the Bayesian Information Criterion values calculated using MEGA7 (Kumar, Stecher, and Tamura 2016). ML phylogenetic reconstruction was performed using PhyML with a GTR nucleotide substitution model and 100 bootstrap replicates. Phylogenetic analysis was also performed using a Bayesian Markov Chain Monte Carlo (MCMC) framework using BEAUti and BEAST v1.10.4 (Suchard et al., 2018) and run via the CIPRES server. Prior to assigning traits to the sequences, marginal likelihood estimation was performed using path sampling/stepping-stone sampling to choose the most appropriate combination of tree model (coalescent constant size or coalescent Gaussian Markov random field (GMRF) Bayesian skyride) and clock models (strict or uncorrelated relaxed) using a GTR nucleotide substitution model (four gamma categories, estimated base frequencies, and no codon partitioning). MCMC outputs from different runs were evaluated and convergence confirmed using Tracer v1.7.1 (Rambaut et al., 2018). A model with a coalescent constant size tree prior and uncorrelated relaxed clock (lognormal distribution) (Drummond et al., 2006) was determined to be the best fit for the data, based on the log marginal likelihood estimates from path sampling/stepping-stone sampling. This model of nucleotide evolution was used for the subsequent analysis of the discrete traits, ‘host category (livestock or wildlife)’, and ‘country’. Asymmetric substitution models were selected for the discrete traits since these are the biologically more plausible scenario of virus transmission. Bayesian Stochastic Search Variable Selection (BSSVS) was also used, which limits the number of rates to those that adequately explain the phylogenetic diffusion process. At least two independent MCMC chains, of 40 million steps each, were performed for each analysis, and Tracer v1.7.1 was used to confirm that the MCMC chains converged at the same level and assess effective sample sizes. LogCombiner was used to combine the output of the independent BEAST runs to generate tree and log files for analysis. MCC trees were generated using TreeAnnotator v1.10.4., and Figtree v1.4.4 was used to visualise and interpret MCC trees and derive TMRCA estimates. Evolutionary rates (ucld.mean) were taken from the combined log files analysed in Tracer v1.7.1. MCC trees and geocoded metadata were imported into Microreact to visualise temporal and geographic spread (Argimon et al., 2016). The MCC tree and log files from the BEAST analysis were uploaded to the SpreaD3 software (Spatial Phylogenetic Reconstruction of EvolutionAry Dynamics using Data-Driven Documents (D3); Bielejec et al., 2016), in order to visualize the output from the BSSVS procedure and compute Bayes factors for transitions. For country transitions, each country was assigned one latitude and longitude coordinate, either the precise sampling location for those countries with a single PPRV sequence in the data set (Kenya, Tibet, Ghana), the GPS location of the saiga3 sample for Mongolia, or the country centroid coordinates (worldmap.harvard.edu) for other countries with >1 sequence.

### 4.6 Detection of selection pressures

Multiple analysis methods were implemented to detect positive selection in our phylogeny. FUBAR (Murrell et al., 2013) and FEL (Kosakovsky Pond and Frost 2005), both available at datamonkey.org (Weaver et al., 2018), were used to detect positive selection at individual sites across the whole PPRV phylogeny. MEME (Murrell et al., 2012) analysis was also performed on datamonkey.org, with the capability to identify sites under episodic selection (i.e. in a subset of branches) as well as under pervasive selection (Murrell et al., 2012; Spielman et al., 2019). In addition, the ratio of non-synonymous to synonymous nucleotide substitutions (*ω* = dN/dS) was estimated for different selection models (in which the *ω* ratio varies among codons) using CodeML as implemented in EasyCodeML (Gao et al., 2019). The model M7 (beta; no positive selection) was compared to the model M8 (beta&ω; positive selection) for each gene using likelihood ratio tests (LRTs) (Nielsen and Yang 1998; Yang et al., 2000) (Table S6). If model M8 was more likely than M7, the Bayes empirical Bayes method (Yang, Wong, and Nielsen 2005) was used to calculate the posterior probabilities for site classes and identify sites under positive selection. Finally, three methods were implemented in datamonkey.org to detect linage-specific selection, using user-defined PhyML ML trees, and alignments for the CDS of individual genes. The monophyletic clade of Mongolian/Chinese sequences was selected *a priori* to test for selection acting on these branches. aBSREL (Smith et al., 2015) was used to detect branches under positive selection. BUSTED (Murrell et al., 2015) was also used to test for gene-wide lineage-specific positive selection. Specific sites under selection in the selected clade were identified using FEL (Kosakovsky Pond and Frost 2005). P/V/C genes were omitted from our selection pressure analyses since these are expressed from overlapping reading frames, which biases the dN/dS estimates and invalidates such analysis (Monit et al., 2015).

### 4.7 Protein homology modelling

The predicted structures of PPRV Hs were modelled by submitting the amino acid sequences of each H to the SWISS_MODEL automated protein structure homology modelling server in ‘alignment’ mode (Schwede et al., 2003). Structures were visualised from the .pdb files using Swiss-pdb viewer.

## Supplementary Material

veab062_Supp

## Data Availability

PPRV genomes have been submitted to GenBank under the following accession numbers: MZ061719, MZ061720, MZ061721, and MZ061722, and the alignment used for analysis is available in supplementary S1 File.
