# Supplementary material for: Molecular epidemiology of peste des petits ruminants virus emergence in critically endangered Mongolian saiga antelope and other wild ungulates
Source: Virus Evol. 2021 Jun 25;7(2):veab062. doi: 10.1093/ve/veab062 (PMC8570150; doi:10.1093/ve/veab062)
Supplement: veab062_Supp [file veab062_supp.zip › Supplementary Figs and Tables.pdf]

S1 Fig

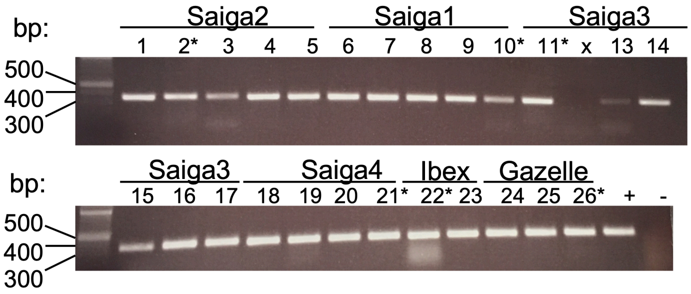

S1 Fig. Molecular detection of PPRV N gene in different tissues from wild Mongolian ungulates. RT-PCR for PPRV N gene was performed on 2ul RNA extracted from different tissue samples from the indicated host (RNA concentration ranged from 26-1125 ng/ul) prior to gel electrophoresis and UV transillumination. Lane numbers refer to sample IDs for different tissues given in S1 Table. DNA ladder markers of different base pair (bp) lengths are shown. ‘x’ denotes an empty lane; ‘-’ denotes a no template control and ‘+’ denotes a positive control RNA template. Samples used subsequently for whole genome NGS are marked with *.

S2 Fig

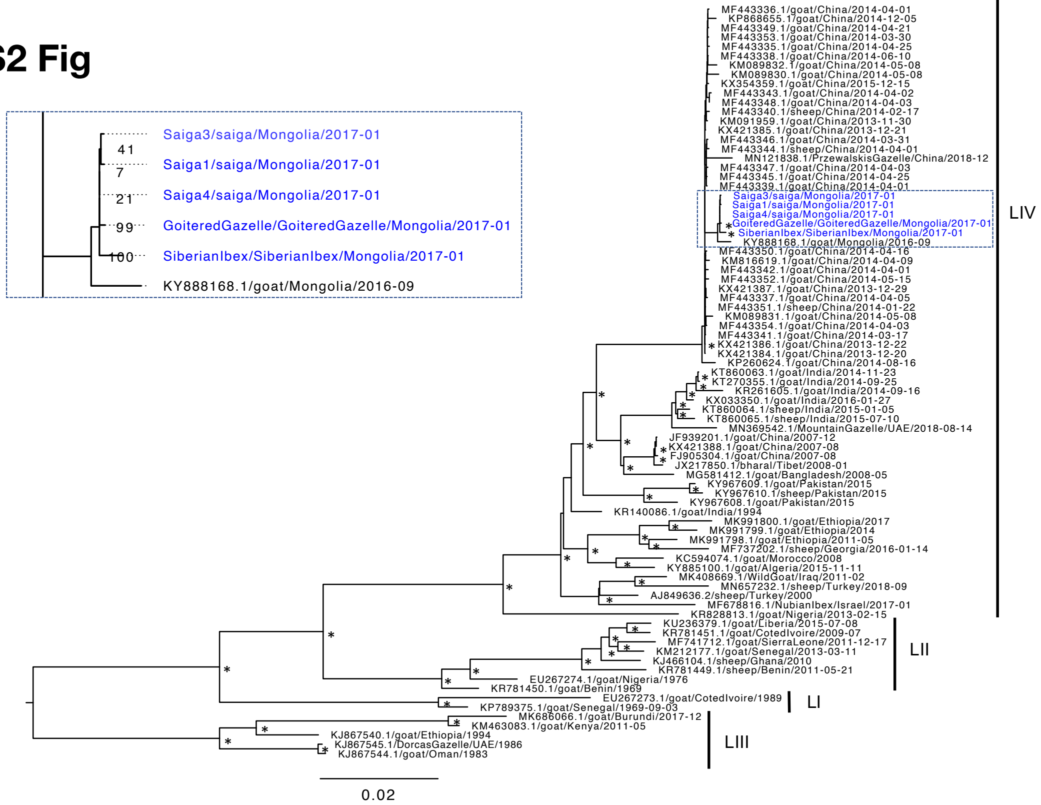

S2 Fig. Maximum likelihood phylogeny of PPRV genomes.

81 PPRV genomes were analysed using PhyML with a GTR nucleotide substitution model and 100 bootstrap replicates. The novel genomes from this study are shown in blue. Lineages, referred to as LI, LII, LIII or LIV, are shown. Scale bar shows nucleotide substitutions per site. * indicates bootstrap proportion > 0.9 at the node opposite. The left-hand panel, demarcated by the dashed box, shows an enlargement of the Mongolian PPRV clade, with bootstrap replicate support shown opposite the corresponding nodes.

S3 Fig

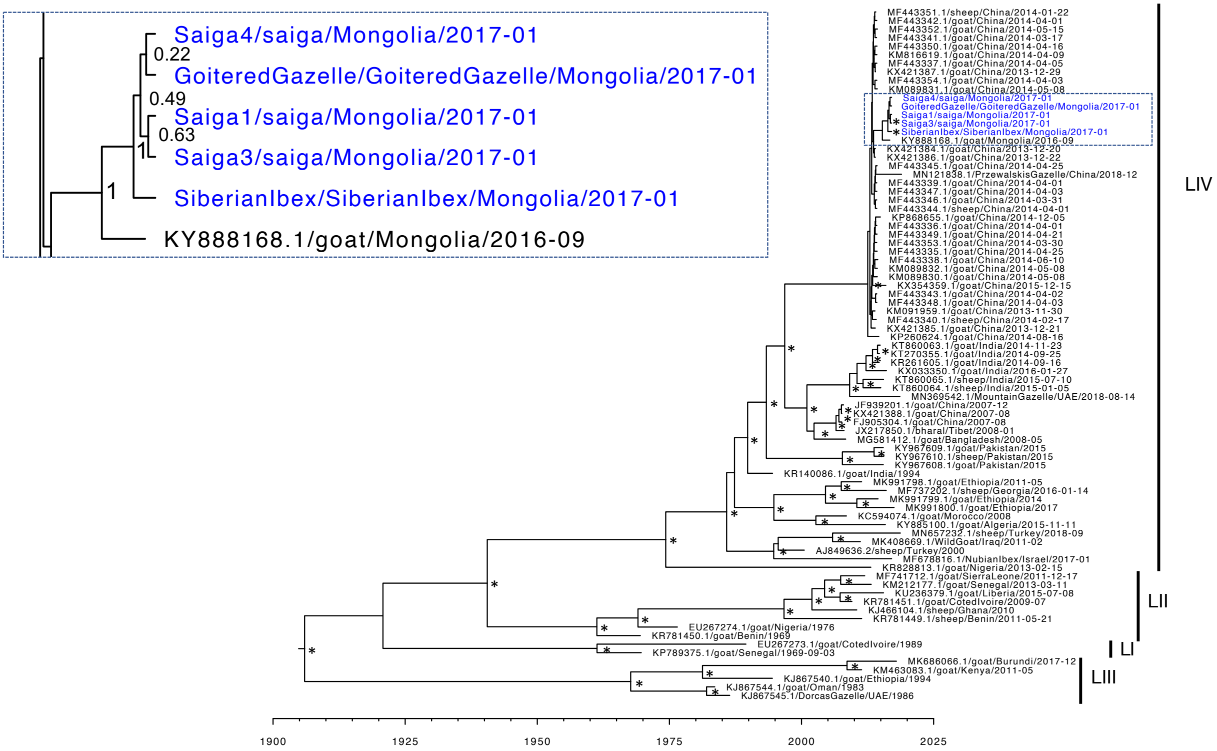

S3 Fig. Untraited Bayesian time-scaled Maximum Clade Credibility Tree.

MCC tree from the combined output of three MCMC chains run in BEAST v1.10.4, inferred without partitioning the data by traits, and visualized in FigTree. The novel genomes from this study are shown in blue. x-axis shows date. Lineages, referred to as LI, LII, LIII or LIV, are marked. * indicates posterior probability > 0.9 at the node opposite. The left-hand panel, demarcated by the dashed box, shows an enlargement of the Mongolian PPRV clade, with posterior probabilities shown opposite the corresponding nodes.

S4 Fig

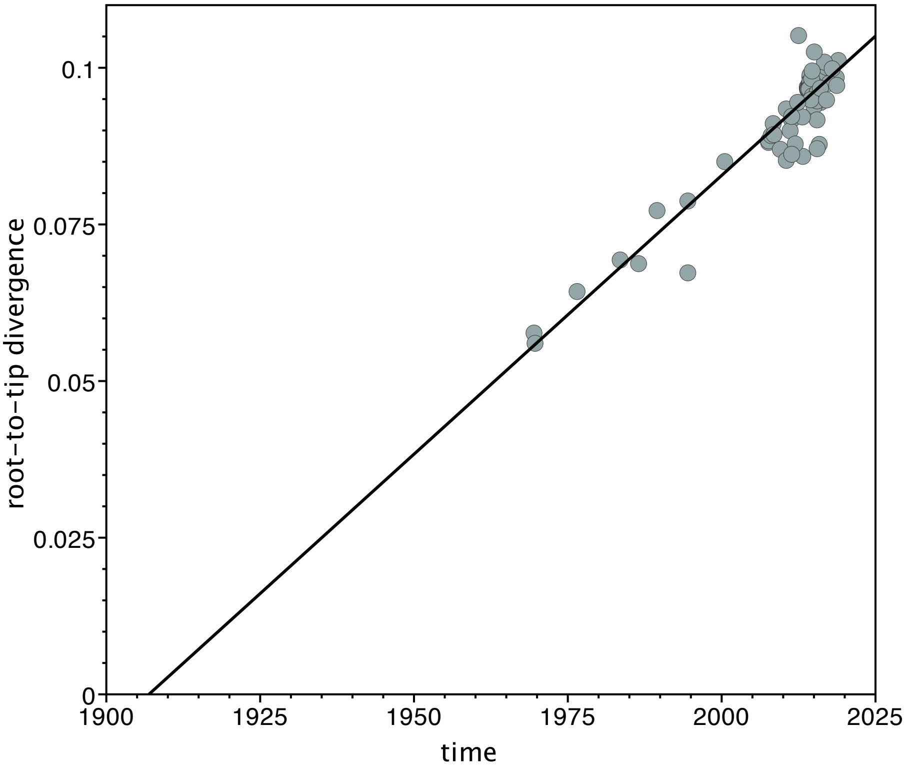

S4 Fig. TempEst analysis of temporal signal in the PPRV genome dataset.
Plot from TempEst showing root-to-tip genetic distance against sampling date for a ML phylogeny of 85 PPRV genomes, using the best-fitting root. The correlation coefficient for the regression was 0.9362 and R^2 was 0.8764.

S1 Table

Host ID	Species Common Name	Sample Collection Date (YYYY-MM)	Province	Soum or subdistrict	Name of sampling location	GPS coordinates		Sample Type (RNA)	Sample ID
						Latitude	Longitude		
Saiga_2	Mongolian saiga antelope	2017-01	Khovd	Chandmani	Takhilt	47.35.55.5	93.13.51.5	lung	1
								liver	2
								spleen	3
								heart	4
								mesenteric lymph node	5
Saiga_1	Mongolian saiga antelope	2017-01	Khovd	Chandmani	Nuramt	48.03.51.3	92.46.15.3	eye swab	6
								gum scurf	7
								palate scurf	8
								nasal swab	9
								tongue scurf	10
Saiga_3	Mongolian saiga antelope	2017-01	Khovd	Chandmani	Suudal khuruu	47.28.09.1	93.30.48.8	spleen	11
								liver	13
								mesenteric lymph node	14
								nasal swab	15
								eye swab	16
								blood	17
Saiga_4	Mongolian saiga antelope	2017-01	Gobi-Altai	Khukhmorit	Sain ust	47.16.03.6	94.07.22.4	spleen	18
								liver	19
								heart	20
								lung	21
Siberian ibex	Siberian ibex	2017-01	Gobi-Altai	Tugrug	Khurengoliin ekh	45.45.21.1	95.11.57.1	liver	22
								eye swab	23
Goitered gazelle	Goitered gazelle	2017-01	Khovd	Darvi	Tungalagiin us	46.48.04.9	93.40.08.0	lung	24
								tongue tissue	25
								soft palate	26

S1 Table. Sampling locations and sample types for PPRV-infected wildlife.
Sample ID refers to lane labels in S1 Fig.

S2 Table

Sample	Total reads	PPRV-specific reads	% PPRV	PPRV genome coverage
Saiga_1	34,322,736	1008	<0.003	x 9.5
Saiga_2	21,266,002	40	<0.0002	N/A
Saiga_3	20,500,220	318,857	1.56	x 2998
Saiga_4	33,389,334	122,645	0.37	x 1153
Goitered gazelle	36,678,534	124,090	0.34	x 1167
Siberian ibex	39,462,531	2,908	0.01	x 27

S2 Table. Illumina NGS read summary for wildlife samples.
Total read number, PPRV-specific read number and % total reads which were PPRV are shown for the five novel PPRV genomes from wildlife hosts. Average genome coverage was calculated as (read count * read length) / total genome size. N/A: not applicable since no PPRV genome was obtained for the saiga_2 sample owing to the very low number of PPRV-specific reads.

S3 Table

Recombinant Sequence	Minor Parental Sequence	Lin-r	Lin-mp	Breakpoint Positions In Alignment		Detection Methods						
				Begin	End	RDP	GENECONV	Bootscan	Maxchi	Chimaera	SiSscan	3Seq
KR828814.1/goat/Nigeria/2012-05-09	KR828813.1/goat/Nigeria/2013-02-15	II	IV	3096	4001	1.38E-55	7.05E-55	1.12E-55	7.39E-14	4.71E-14	2.98E-15	1.37E-11
KR828814.1/goat/Nigeria/2012-05-09	KR828813.1/goat/Nigeria/2013-02-15	II	IV	6388	6848	4.74E-29	1.31E-24	4.81E-19	5.41E-08	2.50E-07	1.22E-06	1.37E-11
KR828814.1/goat/Nigeria/2012-05-09	KJ867541.1/goat/Ethiopia/2010*	II	IV	550	1073	9.97E-13	NS	3.57E-15	3.57E-05	0.033456	NS	8.63E-07
KJ867541.1/goat/Ethiopia/2010	KC594074.1/goat/Morocco/2008*	IV	IV	4138	5544	8.72E-41	1.47E-37	3.72E-39	1.09E-16	3.78E-16	7.72E-21	0.004423
KJ867543.1/goat/Uganda/2012	KC594074.1/goat/Morocco/2008*	III	IV	4110	5556	NS	5.64E-245	1.48E-235	1.54E-44	1.27E-44	9.19E-53	NS
KT633939.1/ibex/China/2015-01-20	KR781450.1/goat/Benin/1969*	IV	II	9084	9556	3.20E-40	1.03E-32	1.56E-38	9.99E-10	5.40E-09	2.71E-09	1.03E-11
KT633939.1/ibex/China/2015-01-20	KR781450.1/goat/Benin/1969*	IV	II	6623	6922	1.14E-21	5.33E-19	1.17E-20	0.00012	0.0009816	4.54E-05	2.16E-10

S3 Table. Recombination analysis using RDP4.

The PPRV genome alignment (n=85) was analysed using Recombination Detection Program v4.101 (RDP4) using default settings. The four genomes unambiguously identified as recombinant sequences are shown, for which at least 5 of 7 of the detection methods found significant evidence of recombination. Recombinant: genome sequence identified as potential recombinant; Lin-r: PPRV genetic lineage of Recombinant; Minor parental sequence: genome sequence identified as most likely minor parent of the recombinant, i.e. most closely related to the genome portion inserted; * indicates that other potential minor parents were also identified by RDP4 for that recombination event; Lin-mp: PPRV genetic lineage of minor parent; Begin: average genome position (in alignment) of the beginning breakpoint point of recombination; End: average genome position (in alignment) of the end breakpoint point of recombination; NS: not significant. The p-values for analysis with each of the 7 different recombination detection algorithms are shown, after Bonferroni correction for multiple comparisons.

S4 Table

Sequence ID/ GenBank Accession No.	PPRV Lineage	Date (YYYY-MM)	Country	Host species (*Latin name*)	Free-ranging/ Captive	Disease event	Key epidemiological & livestock interface data for disease events associated with wildlife-origin PPRV genomes
Saiga_1	IV	2017-01	Mongolia	Mongolian saiga antelope (*Saiga tatarica mongolica*)	Free-ranging	Mass mortality<br><br>>80% population decline estimated	The first suspected (not laboratory confirmed) PPR deaths in saiga were reported by herders in December 2016 before official confirmation of PPRV infection in saiga on 27th December 2016 (Pruvot et al. 2020). The first PPR outbreak in livestock in Mongolia occurred in August 2016 (OIE report 20934). Mass livestock vaccination was undertaken in Western Mongolia in October 2016 (Kock 2017; Pruvot et al. 2020). The last saiga PPR case was reported in May 2017 (Pruvot et al. 2020).
Saiga_3 (MZ061719)	IV	2017-01	Mongolia	Mongolian saiga antelope	Free-ranging	As above	As above
Saiga_4 (MZ061720)	IV	2017-01	Mongolia	Mongolian saiga antelope	Free-ranging	As above	As above
Siberian ibex (MZ061721)	IV	2017-01	Mongolia	Siberian ibex (*Capra sibirica*)	Free-ranging	Mortality (clusters)	Clusters of cases; 24 ibex carcasses disposed by government January-June 2017 (Pruvot et al. 2020). Suspected (not laboratory confirmed) cases reported in ibex in July/August 2016 in South-western part of Khovd province. The latest confirmed ibex case occurred in January 2018.
Goitered gazelle (MZ061722)	IV	2017-01	Mongolia	Goitered gazelle (*Gazella subgutturosa*)	Free-ranging	Mortality (sporadic)	Sporadic cases; 41 Goitered gazelle carcasses disposed by government January-June 2017 (Pruvot et al. 2020). Post-mortem histological findings in PPRV-infected goitered gazelle given in (Pruvot et al. 2020).
MN121838.1	IV	2018-12	China	Przewalski's gazelle (*Procapra przewalskii*)	Free-ranging	Mortality (single case)	Full genome reported by (Li et al. 2019). Outbreak of PPRV in sheep occurred in the same area of northwest Gansu province in October 2018 (Li et al. 2019).
KT633939.1*	IV	2015-01	China	Ibex (*Capra ibex*)	Free-ranging	Mortality (38 ibex mortalities)	Full genome reported by (Zhu et al. 2016). 38 ibex mortalities reported in January/February 2015 (Xia et al. 2016). Mucopurulent oculo-nasal discharge, diarrhoea and pulmonary congestion at post-mortem was reported (Zhu et al. 2016).
JX217850.1	IV	2008-01	China (Tibet)	Bharal (*Pseudois nayaur*)	Free-ranging	Mortality	Full genome reported by (Bao et al. 2012). Sequenced case showed PPR-compatible clinical signs. 19 dead bharal and 6 dead Mongolian gazelles (*Procapra gutturosa*) were found in the same location but PPRV was not confirmed in these (Bao et al. 2011). Epidemiological evidence of PPR outbreaks in livestock nearby in September 2007 (Bao et al. 2011).
MN369542.1	IV	2018-08	UAE	Mountain gazelle (*Gazella gazella*)	Free-ranging	Mass mortality (hundreds of deaths)	Gazelles share desert grazing with free-roaming domestic small ruminants in this area. No concurrent disease was seen in domestic small ruminants.
KJ867545.1	III	1986	UAE	Dorcas Gazelle (*Gazella dorcas*)	Captive	Multi-species disease outbreak	Full genome reported by (Muniraju et al. 2014). Disease outbreak in a zoological collection at Al Ain which clinically affected gazelles (Gazellinae), ibex and sheep (Caprinae) and gemsbok (Hippotraginae) (Furley et al. 1987).
MF678816.1	IV	2017-01	Israel	Nubian ibex (*Capra nubiana*)	Captive	Mortality	Over 2/3 of a captive herd of 32 Nubian ibex died following peracute/acute presentation (Berkowitz et al. 2019). Pathological findings differed from those typical in sheep and goats: abomasitis was seen commonly but oral and pulmonary lesions were rare.
MK408669.1	IV	2011-02	Iraq (Kurdistan)	Wild goat (*Capra aegagrus*)	Free-ranging	Mass mortality	>750 deaths in *Capra aegagrus* were reported August 2010 and February 2011 (Hoffmann et al. 2012). This was the first report of PPRV in Kurdistan but the affected area is close to the Turkish border where PPRV is endemic (Hoffmann et al. 2012). No concurrent disease in domestic ruminants was reported, likely due to annual vaccination (Hoffmann et al. 2012). Due to its close relationship to AJ849636.2/sheep/Turkey/2000, this strain was inferred to have been circulating in the region for more than 10 years (Hoffmann et al. 2012).

S4 Table. Twelve PPRV full genomes available from wildlife host species and associated metadata. The genomes generated in this study are shown in grey. (*): this sequence showed a significant signature of recombination, most likely associated with laboratory contamination, and was not included in this study.

S5 Table

From	To	Bayes Factor	Posterior Probability
China	Mongolia	494	0.96
Ethiopia	Georgia	103	0.82
Kenya	Burundi	65	0.75
Turkey	Iraq	64	0.74
China	Tibet	56	0.72
India	UAE	51	0.70

S5 Table. Bayes factors for spread of PPRV between countries.
Using SpreaD3, Bayes Factors (BFs) were calculated using the log file from the BEAST BSSVS analysis and a discrete set of longitude and latitude coordinates for each country, coupled to a geoJSON formatted world map. The output gave BFs for all possible transitions between locations. The table shows the transitions with posterior probabilities >0.7. The transition from China to Mongolia, which had the highest BF of any transition, is highlighted in grey.

S6 Table

Protein	LnL M7	LnL M8	LRT
**N**	-6569.74	-6554.43	<0.0001
**M**	-4106.74	-4106.74	1.00
**F**	-5750.98	-5749.07	0.15
**H**	-8619.88	-8613.37	0.002
**L**	-25535.46	-25513.47	<0.0001

S6 Table. Values of Log-likelihood (lnL) for PPRV genes using different selection models in the CodeML analysis, and LRT comparing the two models.
Two different site selection models (in which the ω ratio varies among codons) were implemented in CodeML: M7 (beta; no positive selection) and M8 (beta&ω; positive selection). For each gene, Log-likelihood (lnL) values are shown and the likelihood ratio test (LRT) to show the significance of model comparison. Bayes empirical Bayes (BEB) were used to calculate the posterior probabilities for site classes and identify sites under positive selection.

S7 Table

Branch name	LRT	Test p value (corrected for multiple testing)	ω distribution
KP260624_1_GOAT_CHINA_2014_08_16	35.5617	2.91×10^{-7}	ω1 = 0.327 (100%)<br>ω2 = 4150 (0.20%)
KY888168_1_GOAT_MONGOLIA_2016_09	12.3338	0.0330	ω1 = 0.824 (100%)<br>ω2 = 1970 (0.21%

S7 Table: Evidence from aBSREL for episodic diversifying selection acting on the PPRV L gene.
The PPRV L gene was analysed using aBSREL with the China/Mongolia clade selected the set of foreground branches on which to test for episodic diversifying selection. Significance was assessed using the likelihood ratio test statistic for selection (LRT) at a threshold of $p \leq 0.05$, after correcting for multiple testing. The two branches shown were identified as under positive selection, while all other branches were best described by a single ω rate category (ω1). The ω distribution shows inferred estimates for ω1 and ω2 and proportion of sites in each category.
